# MARS1 mutations linked to familial trigeminal neuralgia via the integrated stress response

**DOI:** 10.1186/s10194-022-01537-2

**Published:** 2023-01-14

**Authors:** Anni Wang, Zimu Song, Xu Zhang, LiFei Xiao, Yan Feng, Chong Qi, Guohuan Zhang, Jinbo Bai, Yang Liu, Tao Sun, Fangang Meng, Feng Wang

**Affiliations:** 1 Beijing Neurosurgical Institute, Beijing Tiantan Hospital, Capital Medical University, Beijing, People’s Republic of China; 2 Department of Neurosurgery, Beijing Tiantan Hospital, Capital Medical University, Beijing, People’s Republic of China; 3 Department of Neurosurgery, General Hospital of Ningxia Medical University, Yinchuan, Ningxia People’s Republic of China; 4 School of Medicine, Nankai University, Tianjin, People’s Republic of China; 5 China Ningxia Key Laboratory of Cerebrocranial Disease, The Incubation Base of National Key Laboratory, Ningxia Medical University, Yinchuan, People’s Republic of China; 6 State Key Laboratory of Environmental Chemistry and Ecotoxicology, Research Center for Eco-Environmental Science, Chinese Academy of Sciences, Beijing, People’s Republic of China; 7 Sino-Danish College, University of Chinese Academy of Science, Beijing, People’s Republic of China; 8 Beijing Key Laboratory of Neurostimulation, Beijing, People’s Republic of China; 9 Chinese Institute for Brain Research, Beijing, People’s Republic of China; 10 Department of Neurosurgery, The First Affiliated Hospital, Zhejiang University School of Medicine, Hangzhou, Zhejiang People’s Republic of China

**Keywords:** Familial trigeminal neuralgia, RNA sequencing, Whole-exome sequencing, MARS1, Tyrosyl-tRNA synthetase, Integrated stress response

## Abstract

**Background:**

While new genetic analysis methods are widely used in the clinic, few researchers have focused on trigeminal neuralgia (TN) with familial clustering (≥ 2 TN patients in one kindred family). Previous literature suggests that familial trigeminal neuralgia (FTN) may be associated with inherited genetic factors. To date, few next-generation sequencing studies have been reported for FTN. This study investigated the pathogenic mechanism of FTN by using whole-exome sequencing (WES) technology, which may enhance our understanding of human TN pathophysiology.

**Method:**

We performed WES for 7 probands from families of FTN. Sanger sequencing was performed for two control groups (FTN family members group and nonfamilial TN subject group) to potentially identify new FTN-related gene mutations. In families where FTN probands carried potentially pathogenic gene mutations, the ribonucleic acid (RNA) of FTN probands and related family members, as well as nonfamilial TN patients were analysed by RNA sequencing (RNA-seq) to confirm differential gene expression.

**Results:**

Seven probands were derived from 3 Chinese families. WES and Sanger sequencing identified MARS1 mutation c.2398C > A p.(Pro800Thr) in Family 1. MARS1 mutation was confirmed in 14/26 [53.8%] members of Family 1 in FTN family member group, while none of nonfamilial TN subjects had this MARS1 mutation. RNA-seq showed that 3 probands in Family 1 had higher expression of Fosl1 (Fos-like antigen 1) and NFE2 (Nuclear factor, erythroid 2) than 3 subjects in the nonfamilial TN subject group. Fosl1 and NFE2 are genes related to integrated stress response (ISR).

**Conclusion:**

MARS1 mutations may cause chronic activation of ISR, contribute to ISR pathophysiological changes in FTN, and cause/accelerate peripheral nerve degeneration. The findings of this study can enrich our knowledge of the role of molecular genetics in TN in humans.

## Background

Trigeminal neuralgia (TN) is the most common, severe form of neuropathic pain, and TN is typically characterized by recurrent brief, severe paroxysmal pain attacks within the distribution of one or more branches of the fifth cranial nerve (trigeminal nerve) [1]. Most idiopathic TN cases are sporadic, with occasional familial clustering. TN affects approximately 3–4 per 100,000 people worldwide, and familial cases account for 2–5% of these cases [2].

The underlying pathophysiology of TN is not fully understood. The main hypothesis of TN pathophysiology is that the compression from a blood vessel causes focal demyelination of the trigeminal nerve at the root entry zone [3]. Clinical evidence for the causality between neurovascular compression and TN has been supported by data documenting demyelination at the compressed site. However, the following several situations have not been explained: (i) the symptoms of a significant subset of TN patients recur after microvascular decompression (MVD); (ii) vascular compression of the trigeminal nerve is observed in 13% to 58% of asymptomatic individuals; and (iii) at the time of surgery, a significant proportion of TN patients demonstrate minimal demonstrable vascular compression [4].

Multiple FTN studies have shown that the onset of TN occurs earlier in patients with FTN than in those with sporadic TN [5, 6]. The involvement of genetic factors in the pathogenesis of FTN has been suggested. In particular, a recent study revealed rare gene mutations in ion channels in patients with a family history of TN, but the frequency and clinical significance of this finding are unknown [7–9]. Although the role of genetic factors in the development of TN, particularly in familial TN is obscure, it was suggested to be more prominent than previously hypothesized [4]. Therefore, it is necessary to carry out genetic research on FTN and TN.

Technological advances in the usability of whole-exome sequencing (WES) now offer unprecedented opportunities for simultaneous high-throughput investigations of genes [10, 11]. In the past decade, ribonucleic acid sequencing (RNA-seq) has become an integral part of the transcriptome-wide analysis of differential gene expression [12]. RNA-seq approaches are used for studying many different aspects of RNA biology, including RNA structure (the structurome), differential gene expression, and translation (the translatome). Nevertheless, few researchers have focused on FTN. The lack of WES and RNA-seq studies on FTN may lead to a knowledge gap in the understanding of the molecular genetics of TN, and such a knowledge gap is a fundamental obstacle for targeted therapeutics.

Here, we used WES and RNA-seq to analyse a group of patients with FTN, and this exploratory genetic analysis provides a reference for more genetic studies with more patients, unrelated controls, and healthy familial members in the future. Furthermore, the findings of this study may improve the understanding of human TN pathophysiology and promote the development of an animal model of TN.

## Methods

### Participants

The study was approved by the Ethics Committee of the General Hospital of Ningxia Medical University (Number: KYLL-2021–417), and each participant provided written informed consent.

We prospectively screened consecutive patients admitted to the Department of Neurosurgery at the General Hospital of Ningxia Medical University from June 2018 to July 2021. The inclusion criteria in this study were (i) ≥ 2 trigeminal neuralgia patients in one family and (ii) a definite diagnosis of TN according to two or more clinicians (fulfilling the standard for TN according to the third edition of the International Classification of Headache Disorders, ICHD-III). The exclusion criteria were (i) a diagnosis of secondary TN and (ii) a diagnosis of orofacial pain other than TN.

### Control groups

Blood samples from 2 control groups were used in the study. The first, designated the FTN family member group, was composed of blood-related family members who were carefully assessed to exclude any individuals with chronic orofacial pain. The second control group, designated nonfamilial TN subject group, was composed of individuals with chronic orofacial pain and definite diagnosis of TN but were not blood-related family members.

### Clinical assessments

All individuals were interviewed by 2 of the investigators. Forty-seven participants were interviewed regarding symptoms of facial pain and underwent a brief neurologic examination. This examination included strength grading using the United Kingdom Medical Research Council grades and pain measurement using the Visual Analogue Scale (VAS) and the Barrow Neurological Institute (BNI) pain scale. Systematic surveys were conducted using a dedicated questionnaire, which included questions on demographic data and clinical characteristics, such as age of onset, details on triggers, and possible concomitant continuous pain.

### Blood sample collection

Eventually, we identified 3 families from Northwest China. Seven TN patients with a family history agreed to undergo venous blood sample collection. From the control groups of FTN family members and nonfamilial TN subjects, we obtained blood samples from 33 and 7, respectively. For all three groups, the blood samples were collected in the morning before breakfast. In addition, the blood samples from the FTN group and nonfamilial TN subject group were collected before medication and after TN attack. All genomic DNA samples from patients and control individuals were extracted from peripheral blood using standard practices.

### Whole-exome sequencing

To identify novel genetic factors that could potentially underly the development of FTN, blood samples from probands were used for WES. The genealogical positions for the probands and FTN family members were determined.

Genomic DNA was extracted from the whole blood of the patients using a TIANamp Blood DNA Kit #DP348-03 (TIANamp, Wuhan, China).

DNA samples were prepared using the xGen Exome Research Panel v1.0(Integrated DNA Technologies, Inc., USA). Sequencing was performed using Illumina HiSeq 2500 (San Diego, CA). The obtained reads were compared with the human genome reference (UCSC hg 19, https://genome.ucsc.edu/) using BWA (Burrows-Wheeler. Aligner), and reads that could not match the reference genome were removed for subsequent analysis (approximately 99.5% of the reads could be analysed).

Mutations were further filtered against more than 20,000 Genomes for all probands, removing mutations with a minor allele frequency > 1%. All mutations detected in patients were checked against the gnomAD (Genome Aggregation Database http://gnomad.broadinstitute.org), ESP (Exome Sequencing Project https://evs.gs.washington.edu/EVS/), ExAC (the Exome Aggregation Consortium (http://exac.broadinstitute.org/), and 1000 Genomes (https://www.internationalgenome.org/) databases. For in silico functional predictions, we used the GERP +  + program (http://mendel.stanford.edu/SidowLab/downlods/gerp/index.html), SIFT (http://sift.jcvi.org), MUpro (http://www.ics.uci.edu//~baldig/mutation), MutationTaster (http://www.mutationtaster.org/), CADD (https://cadd.gs.washington.edu/), and PolyPhen-2 (http://genetics.bwh.harvard.edu/pph2/). At least three or more predictors were used to evaluate deleterious mutations. By assessing the position of the proband in the family diagram, we analyzed the potential mode of inheritance and then screened out the candidate genes. Finally, Mutations were classified using the American College of Medical Genetics and Genomics/Association for Molecular Pathology (ACMG/AMP) criteria as pathogenic (P) or likely pathogenic (LP). Mutations that satisfied the above partial criteria are listed in Table 3. A mutation must satisfy all of the above criteria to be considered pathogenic.

### Sanger sequencing

DNA from probands who carried candidate pathogenic genes and their family members was analysed by Sanger sequencing to validate the WES readings. Two pairs of primers were designed using Oligo 7 and synthesized by the dideoxy method to verify the candidate genes.

### RNA sequencing

RNA samples of probands who carried candidate pathogenic genes and their family members and those from the TN patients were analysed by RNA-seq. All blood samples were homogenized and lysed in TRIzol (Total RNA Extractor) reagent. The quality and concentration of RNA samples were determined by using the Beckman AMPure XP system RNA 6000 Nano LabChip assay of the Agilent 2100 Bioanalyzer system (CA, USA). We first prepared the library for transcriptome sequencing. Briefly, mRNA was isolated from total RNA by using oligo-dT magnetic beads, and then first- and second-strand cDNA synthesis, cDNA purification, and PCR amplification were performed. The sequencing libraries were prepared using the AMPure XP system (Beverly USA). The library was tested to ensure quality using Agilent 2100 Bioanalyze and qRT-PCR (quantitative real-time PCR) according to the manufacturers’ instructions. After library preparation and qualification, the library was sequenced by an Illumina NovaSeq 6000. Next, after data quality control and comparison with the reference genome, we performed multiple analyses such as gene differential expression and gene enrichment.

## Results

### Study population

The demographic details of the probands are presented in Table 1, and those of the two control groups (the FTN family member group and nonfamilial TN subject group) are presented in Table 2. We ultimately included 7 probands, 33 subjects from the FTN family member group, and 7 subjects from the nonfamilial TN subject group in our study. According to the criteria in ICHD-III published in 2018, both the nonfamilial TN subjects group and probands were considered to have classic trigeminal neuralgia [13]. In this study, there were more men than women in all three study groups. There were no sex or VAS score differences between the probands and nonfamilial TN subject group. However, the age of onset was significantly higher in the nonfamilial TN subject group than in the probands (*p* < 0.01).

**Table 1:** The Demographic data and baseline characteristics of probands

Variable	Patient	Sex	Age(y)	Age of onset(y)	Symptomatic site	Therapeutics	BNI	VAS	MRI feature	Surgery feature	BNI (at least 6 m after surgery)	VAS (at least 6 m after surgery)	Complications	Follow up(mo)
Family 1	II-1	F	59	42	Left, V2 and V3	Carba	III	7	The left superior cerebellar artery is closely related to the trigeminal nerve	NA	NA	NA	NA	12
	II-2	M	53	44	Left, V2 and V3	Carba, Gaba, Oxcarba	IV	8	The left superior cerebellar artery is closely related to the trigeminal nerve	The superior cerebellar artery compresses the trigeminal nerve root	II	4	Infection, dizzy	12
	II-3	M	51	32	Left, V2 and V3	Carba	V	9	The left superior cerebellar artery is closely related to the trigeminal nerve	The superior cerebellar artery compresses the trigeminal nerve root	I	2	Infection	18
Family2	II-1	F	53	36	Left (dominant) and right, V2(left) and V2(Right)	Carba	IV	7	Bilateral trigeminal nerves are close to cerebellar artery	Severe compression at the root entry zone of the trigeminal nerve by the superior cerebellar artery	II	5	Dizzy	17
	II-2	F	50	37	Right, V2	Carba	V	9	Bilateral trigeminal nerves are close to cerebellar artery	The superior cerebellar artery compresses the trigeminal nerve root	I	2	NA	9
Family3	I-1	F	71	70	Right, V3	Carba	IV	8	The right superior cerebellar artery is closely related to the trigeminal nerve	NA	I	1	NA	6
	II-2	F	44	34	Left, V3	Carba	III	6	The superior cerebella artery located on the inside of the trigeminal root	NA	NA	NA	NA	6

*V1* Ophthalmic, *V2* Maxillary, *V3* Mandibular afferent fibre, *BNI* Barrow Neurological Institute, *VAS* Visual Analogue Scale, *Carb* Carbamazepine, *Gaba* Gabapentine, *Oxcarb* Oxcarbazepine, *NA* Not available, *m* months

**Table 2:** The demographic data and baseline characteristics of control groups

Variable	FTN family member group, *N* = 33	nonfamilial TN subject group, *N* = 7
Age(TN,y)	36.97	58
Sex (Female/male)	21/12	4/3
Age of onset (TN,y)	NA	54.71
Symptomatic site(TN)		
V1	NA	0
V2	NA	2
V3	NA	5
High blood pressure	4	3
Diabetes	1	0
Lumbar disc protrusion	3	0
Epilepsy	1	0
Therapeutics		
Carbamazepine	NA	7
Gabapentin	NA	1
Other medications	NA	2
No medication	NA	0

*V1* Ophthalmic, *V2* Maxillary, *V3* Mandibular afferent fibre, *NA* Not available, y years

### Probands

Seven probands from 3 northwestern Chinese families were analysed by WES (2 males and 5 females, mean ± SD: age of onset 42.14 ± 12.99 years). The duration of disease ranged from 1 to 19 years (mean ± SD, disease duration 12.28 ± 6.24 years). In terms of therapy, all of the probands had received at least one previous medication for TN after diagnosis. The most common medication was carbamazepine, which partially controlled the attacks. Four of the probands underwent MVD. One of the probands underwent percutaneous balloon compression (PBC). At the follow-up 6 months after the operation, 3 (60%) of 5 probands were pain-free without medication (BNI grade I), 2 (40%) were pain-free without medication (BNI grade II), and the average VAS score without medication was 2.80 ± 1.64 (mean ± SD). All probands underwent WES for analysis.

**Fig. 1:**
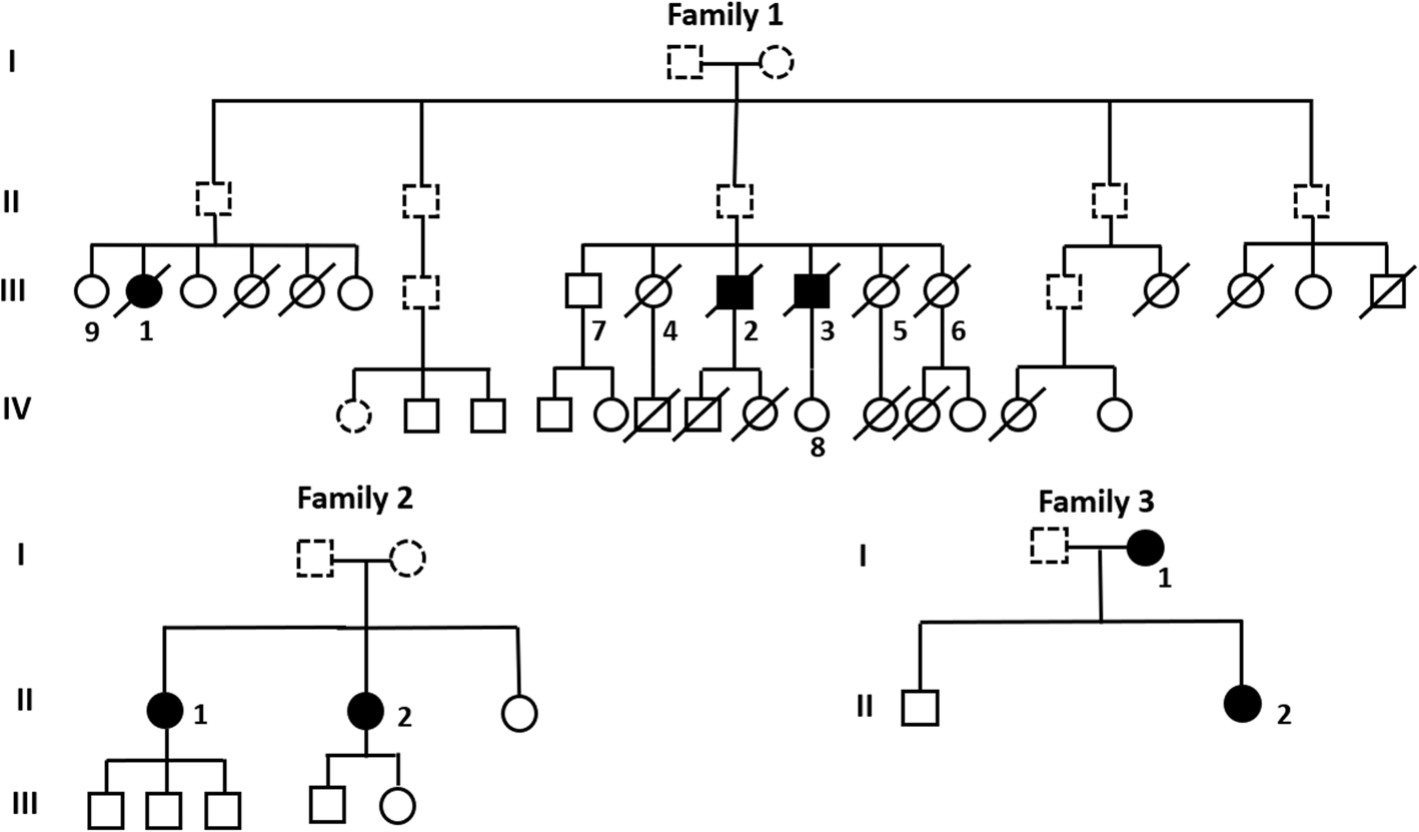
Pedigrees of 3 FTN families. Pedigrees of 3 TN families are shown. Squares indicate male family members and circles indicate female family members. A solid black circle or square denotes the diagnosis of TN. Roman numerals denote generations. The soulid line indicates that blood samples were collected and Sanger sequencing was performed, and the dotted line indicates that blood samples could not be collected for various reasons, such as death. Arrows indicate the confirmed MARS1 mutation (c.2398C > A) by Sanger sequencing. In Family 3, II-2, the proband, had a MARS mutation (c.2104C > T). The subject in Family 1 marked with numbers shows the result of Sanger sequencing, which is shown in Fig. 2

Three probands from the same family (Family 1) presented highly consistent electric shock-like pain in the left maxillary and mandibular that was triggered while eating or brushing their teeth (Fig. 1). Clinical examination demonstrated that all of the patients from Family 1 had hyperesthesia in the left maxillary and mandibular (V2, V3). Two of these patients received MVD treatment due to poor drug tolerance. However, the TN attacks of 1 proband recurred 18 months after the operation, and carbamazepine controlled the painful attacks.

Two probands from a family (Family 2) were sisters, who demonstrated pain attacks on different sides (Fig. 1). The elder sister suffered from electric shock-like pain in the left V2 division when talking, eating, or touching her upper lip, but the pain later spread to the right V2 division. The TN of the younger sister began with paroxysmal pain in the right V2 division. These 2 probands were treated with MVD. The elder sister received left MVD treatment, as her left-side pain most affected her quality of life.

Two probands from another family (Family 3) were the mother and daughter (Fig. 1). Both of these probands presented classic TN. The mother suffered from electric shock-like pain on the right side when eating, drinking, or brushing her teeth. Despite the duration of TN being only 1 year, PBC was performed because drugs only partially controlled her painful attacks. The daughter suffered from paroxysmal pain that was triggered by talking, eating, drinking, or gentle touching of her face, and carbamazepine controlled her painful attacks.

### Control groups

The FTN family member group consisted of 33 family members of the probands. The average age of this group was 36.96 ± 11.23 (mean ± SD), and 26 (78.8%) subjects were members of Family 1. One member from Family 1 had epilepsy, which was well controlled.

Of the 7 subjects included in the nonfamilial TN group, all were considered to have classic TN according to 2 clinicians. The average age of the nonfamilial TN subjects was 58 ± 11.23 (mean ± SD). The age of TN onset was 54.71 ± 10.73 (mean ± SD), which was significantly higher than that of the probands. All of the subjects in nonfamilial TN group had received MVD treatment when the recommended dose of the drug was insufficient for symptom control. After the MVD operation, the average VAS score without medication was 3.29 ± 2.36 (mean ± SD).

### WES and Sanger sequencing

**Fig. 2:**
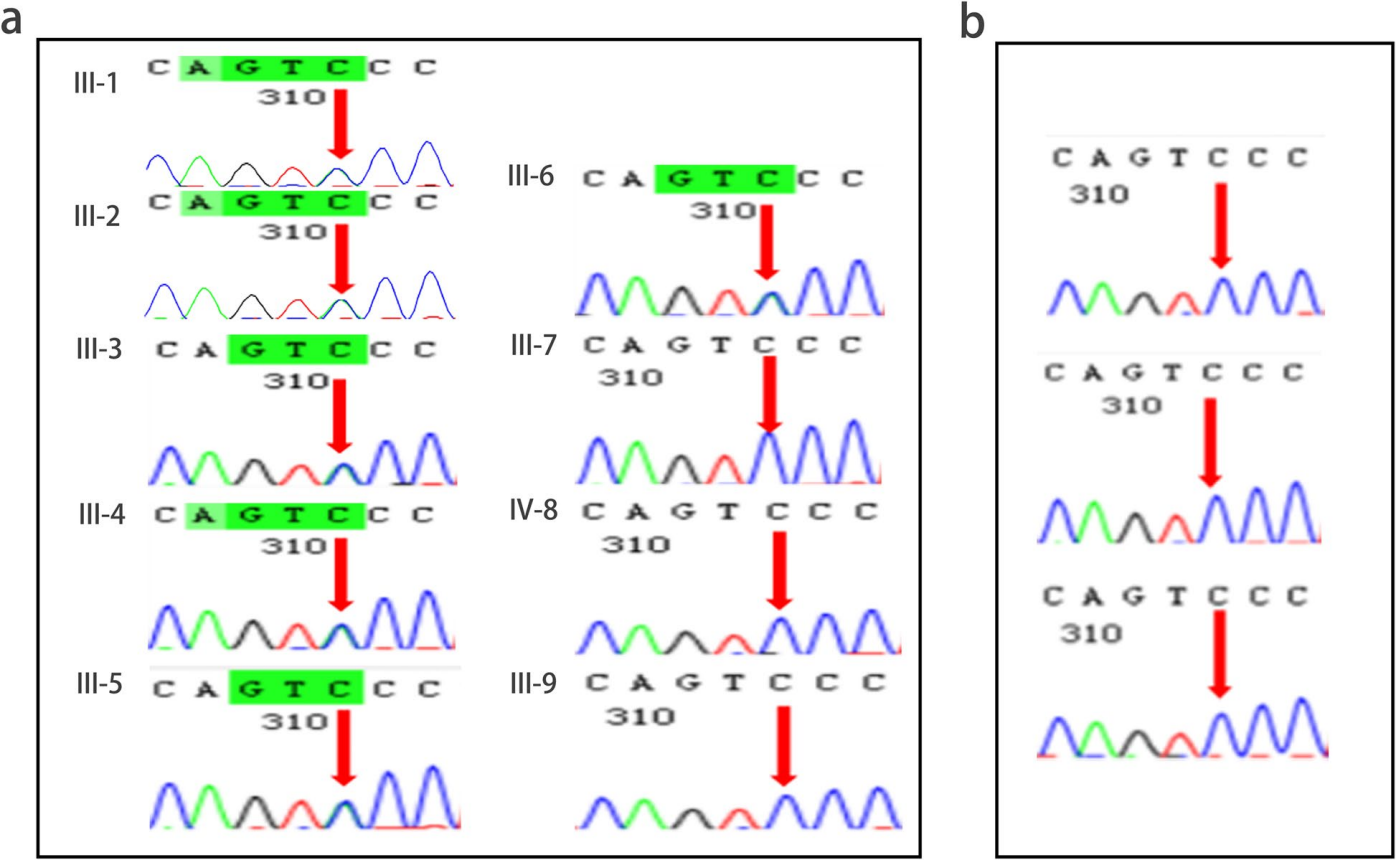
Sanger sequence MARS1 in Family 1 and nonfamilial TN subject group. **a** We performed Sanger sequencing to confirm the MARS1 mutation and found that its presence was widespread in Family 1. Roman numerals denote generations. Numbers 1–3 are probands in Family 1. Numbers 3–9 are subjects in the FTN family member group in Family 1. The MARS1 mutation is identified in numbers 3–6. Numbers 6–9 are negative. **b** We performed Sanger sequencing to confirm the MARS1 mutation (c.2398C > A) in nonfamilial TN subject group. Three subjects did not have MARS1 mutation. All the probands and subjects in Fig. 2 were subjected to RNA-seq

Whole-exome sequencing was performed for 7 probands (Table 3). After systematically filtering the WES data from 7 probands from 3 Chinese families, we identified a mutation shared by the 3 probands from Family 1 that was located in neurodegenerative disorder-related and pain-related genes. As a result, we expanded our analyses in Family 1 by performing Sanger sequencing, and we identified the MARS1 mutation (OMIM: 156560) c.2398C > A p.(Pro800Thr), which was widespread in this family (Sanger sequencing identified the MARS1 mutation in 14/26 [53.8%] members of Family 1 in FTN family member group) (Fig. 2a). In the nonfamilial TN subject group, 7 subjects underwent Sanger sequencing, and none of these subjects had this MARS1 mutation (Fig. 2b). In silico analysis predicted that the MARS1 mutation was likely to be pathogenic (GERP score: 5.06, SIFT score: 0.01, Polyphen2 score: 0.866, PhyloP score: 6.685). According to the ACMG/AMP criteria, this mutation was classified as LP [14]. A MARS1 mutation, c.2104C > T p.(Arg702Trp), was identified in one proband in Family 3, and in silico analysis predicted this mutation to be pathogenic (GERP score: 4.19, SIFT score: 0.00, Polyphen2 score: 0.999, PhyloP score: 9.55). According to ACMG/AMP classification, this mutation was a variant of uncertain significance (VUS). However, this mutation was not widespread in Family 3 according to current data. This might be related to the small sample size in Family 3. Notably, both missense MARS1 mutations (c.2398C > A, c.2104C > T) had an extremely low frequency in the gnomAD, ESP, ExAC, and 1000 Genomes databases.

**Table 3:** All variants detected in our ion channel gene panel from 7 cases with FTN

Family and Patient No.	Genes	Mutations			In silico analysis				
		Nucleotide	Amino acid	Domain	GERP	SIFT	Polyphen2	PhyloP	CADD
Family1 Patient II-1, II-2, II-3	MARS1	c.2398C > A	p.P800T	Anticodon binding	5.06	0.01	0.866	6.685	32
Family1 Patient II-2, II-3	POLG	c.1235C > T	p.P412L	Mitochondrial DNA polymerase	5.55	0.00	0.998	9.624	34
Family1 Patient II-1	SH3TC2	c.2939G > T	p.C980F	Protein expressed	5.37	0.00	0.905	4.868	25.3
Family1 Patient II-1	ACVRL1	c.663G > T	p.W221C	Cell-surface receptor	4.85	0.00	0.997	9.998	33
Family2 Patient II-1, II-2	AMACR	c.844G > C	p.E282Q	Mitochondrial and peroxisomal enzyme	5.7	0.10	0.858	7.611	25.6
Family2 Patient II-1	COL4A1	c.125 A > G	p.H42R	Collagen type IV	3.97	0.37	0.067	2.209	19.57
Family2 Patient II-2	MFN2	c.2162 T > C	p.I721T	Mitofusins	5.28	0.04	0.517	5.949	24.7
Family3 Patient I-1	MARS1	c.2104C > T	p.R702W	Anticodon binding	4.39	0.00	0.997	5.081	7.63
Family3 Patient I-1	SCN10A	c.2161C > T	p.P721S	Ion transport domain	4.19	0.00	0.999	9.55	6.16
Family3 Patient I-1	PINK1	c.C736C > T	p.R246X	threonine protein kinase	5.75	0.00	0.999	6.55	6.79

### RNA sequencing

**Fig. 3:**
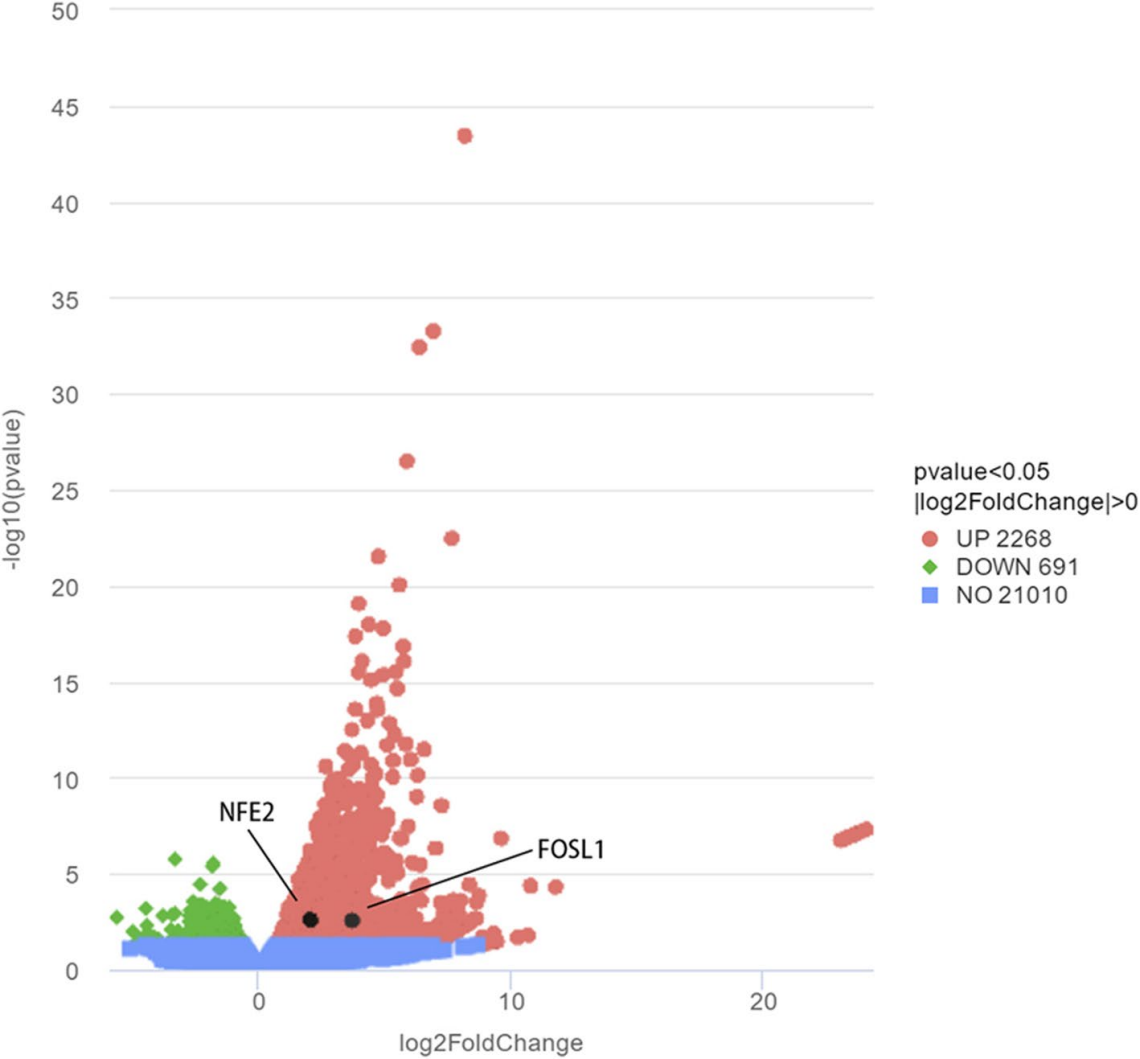
Comparison of 3 probands in Family 1 and 3 subjects from nonfamilial TN subject group. We performed RNA-seq to identify differential gene expression between the blood samples of 3 probands in Family 1 and 3 subjects from the nonfamilial TN subject group. A total of 2268 genes were upregulated (log Fold Change > 0; *p* value < 0.05) and 691 genes were downregulated (log Fold Change < 0; *p* value < 0.05)

We performed RNA-seq for 3 probands with MARS1 mutation (c.2398C > A) in Family 1, 6 subjects from Family 1 in FTN family member group (3 subjects with the MARS1 mutation, and 3 subjects without the MARS1 mutation), and 3 subjects from the nonfamilial TN group. We focused on genes related to the integrated stress response (ISR), which was recently reported to be associated with neurodegenerative diseases and aminoacyltransfer RNA synthetase (AARS) genes. RNA-seq data from ISR-related genes (genes from the Gene Ontology database) demonstrated that Fosl1 and NFE2 expression was higher in the probands in Family 1 than that of nonfamilial TN subject group (Fig. 3) (Table 4). However, there was no significant difference in the expression of ISR-related genes when the probands in Family 1 were compared with the 6 subjects from the FTN family member group.

**Table 4:** Differentially gene expression in ISR-related gene (according to Gene Ontology) in probands in Family 1 and nonfamilial TN subject group

Gene Name	Log2FoldChange	*P* value	padj
EIF2AK1	1.03894837790766	0.096828633331029	0.344759411367066
EIF2AK4	-0.262713965370823	0.568591163947201	0.794701577372947
EIF2S1	-0.609931617812125	0.27501505525723	0.568845377790338
OMA1	-0.323658803329491	0.695631062979278	0.865626557540739
QRICH1	-0.0488001518329257	0.9187313712068	0.966400677485528
NFE2L1	0.0961019183611657	0.805058848901278	0.918692196886347
BATF2	-0.129037608908874	0.882660902672812	0.953343139201189
IMPACT	-0.113595209094019	0.852943893022315	0.940343158206738
BANF1	0.911012614039092	0.0556555566998596	0.257725731564309
NFE2	2.02254584382766	0.00252426514886172	0.0359891080974641
DELE1	-0.632336766467614	0.117271781167043	0.37872479982454
CEBPE	0.443027378386665	0.547726925858066	0.779776141391471
CREBZF	-0.598895902468954	0.277614421747625	0.570823523123742
EIF2AK3	0.0391726592958066	0.944348506943016	0.975925524642364
NFE2L2	-1.00505483777781	0.0947272536106541	0.34064138504051
CREB3	-0.0636740029186381	0.907976631531862	0.962309594677198
ATF4	0.00563961487371987	0.986079565640816	0.994107842161989
BATF3	0.854983432012971	0.425299427295654	0.698933975225451
FOS	-1.63532440986181	0.0393840390145803	0.211113195092196
FOSL1/Fra1	3.68441363042664	0.00271156062275735	0.0377494653327718
PPP1R15A	0.431246476505893	0.353488440812154	0.640913604370964

## Discussion

In this case series, we performed a clinical genomic study of TN with familial clustering. In this study, we found for the first time an important subset of patients with FTN in Northwest China. By using WES and Sanger sequencing, we identified multiple MARS1 mutations that may drive FTN pathogenesis, and this gene has not previously been reported to be related to FTN. Moreover, by using RNA-seq, we analysed ISR-related genes to confirm their differential gene expression, and we found that Fosl1 and NFE2 expression was significantly higher in probands (Family 1) than in nonfamilial TN subject group. Our findings suggest that MARS1 mutations may cause the chronic activation of ISR, contribute to its pathophysiological changes and cause/accelerate peripheral nerve degeneration, which may explain the early age of onset in FTN. The results presented here provide a basis for further investigation into FTN and enrich our understanding of the human molecular genetics of TN.

### Characteristics of FTNs

The occurrence of FTN is not an accidental event. From the data, it is easy to see that the age of onset in probands was more than ten years earlier than that in nonfamilial TN subject group. Notably, this finding is highly consistent with those of other studies [6, 15, 16]. This evidence suggests that genetic factors may be involved in the mechanism of TN pathophysiology and accelerate TN onset.

### MARS

In all probands in 3 families, one missense mutation (c.2398C > A) was screened out so we focused on this mutation. This mutation had a high conservation score and it was classified as LP according to ACMG/AMP guidelines (Table 3), and it exhibited incomplete dominance in this family. MARS1 belongs to the aminoacyl-tRNA synthetase (AARS) gene group [17]. Aminoacyl-tRNA synthetases are important for protein synthesis [18]. He et al. also suggested that aminoacyl-tRNA synthetases sense amino acid levels, transmit amino acid signals to signalling networks, and regulate various cellular functions [19]. To our knowledge, our study is the first to identify MARS1 transmission in FTN patients.

MARS is encoded by nuclear genes, of which there are two subtypes. The gene products of MARS are vital for the translation initiation of mRNAs. MARS1 gene mutations were shown to cause Charcot-Marie-Tooth disease Type 2u (CMT2u), which is a phenotype of this autosomal incomplete dominance neurodegeneration disease according to previous studies [20]. MARS2 mutations were shown to cause autosomal recessive spastic ataxia with leukoencephalopathy (ARSAL), which is also a type of neurodegeneration disorder [21]. Mutations in MARS2 lead to elevated levels of oxidative stress, impaired mitochondrial activity, and neurodegeneration. Plum et al. found that reduced MARS2 levels in Parkinson’s disease brain tissue lead to neurodegeneration [17]. Mutations in all types of MARS are closely related to neurodegenerative diseases. A recent study suggested that AARS-related genes cause deficient myelination [22]. Notably, neurodegeneration caused by demyelination is the core assumption of the pathophysiology of TN [3, 15]. All evidence indicated a close relationship between AARS-related genes and TN under some conditions.

### ISR

Recently, Burgess et al. suggested that mutant tRNA synthase can activate the ISR and lead to neurodegeneration [23]. Moreover, the transgenic overexpression of a tRNA synthase rescued ISR activation in the mouse models of tRNA synthetase mutation of this gene [24]. The ISR is the central regulator of protein homeostasis and is activated in a wide range of neurodegenerative disorders of the brain to respond to stress conditions [25–27]. ISR activation was demonstrated in postmortem brains of individuals and animal models of cognitive and neurodegenerative diseases, including Parkinson’s disease, Alzheimer’s disease, CMT, etc. In particularly, myelin cells from the central or peripheral nervous system synthesize a large number of myelin lipids and proteins and accumulate misfolded or unfolded proteins to activate the ISR in myelination disorders [28, 29]. Thus, MARS1 is a type of tRNA synthase gene, and MARS1 mutations may cause ISR activation to drive neurodegenerative diseases.

The central regulatory factor of the ISR is eukaryotic initiation factor 2 (eIF2) [28, 30]. eIF2 phosphorylation triggers the translation of specific mRNAs, including key transcription factors, such as activating transcription factor 4 (ATF4). Notably, in our study, we performed RNA-seq, and the results showed that Fosl1 and NFE2 expression was higher in probands in Family 1 than individuals in the nonfamilial TN subject group. Fos1 and NFE2 are ATF4-interacting partners [31–33]. A previous study suggested that a high level of NFE2 increases the expression of ATF4 and thus activates the ISR [34]. The binding of Fos1 and ATF4 as dimers plays an important role in a variety of mechanisms in transcriptional regulation [32]. Therefore, we suggest that the MARS1 mutation found in the probands may cause ISR activation which leads to TN.

We also performed RNA-seq for the probands in Family 1 and their family members in the FTN family member group. However, there was no significant difference in ISR-related genes between the 2 groups, regardless of whether FTN family members carried the MARS1 mutation. This may be related to the difference in age and the small sample size of probands and FTN family members.

### The relationship between FTN and CMT

CMT is a common hereditary peripheral neuropathy. To date, at least 25 genes have been identified to be associated with CMT. The core of CMT pathology is demyelination (CMT type 1) and axonal degeneration (CMT type 2) [35, 36]. Interestingly, accumulating evidence indicates a potential link between FTN and CMT. To date, at least 9 CMT families have FTN (Table 5). Several studies identified potential gene mutations in probands from FTN families [5, 6, 16]. These mutated genes are the confirmed pathogenic genes of CMT.

**Table 5:** Review the literature of FTN with CMT

Year	Reference	CMT Affected Members	Type of CMT	Sex(M/F) for CMT	TN Affected Members	Sex(M/F) for TN	Age of TN onset (min–max)	Other Cranial Nerve Involvement	Treatment	Probably related genes
2000	Marcus de Matas et al. [37]	2	NA	2/0	2	2/0	24–41	NA	NA	NA
2004	Todd R. Aho et al. [38]	3	CMT1A	2/1	3	2/1	NA	NA	NA	PMP22
	Robert J. Coffey et al.	8	NA	3/5	4	1/3	39-?	NA	NA	NA
1977	Cruse, RP et al. [39]	13	CMT1	6/7	6	More than 4 females	NA	Hearing loss Demyelinating with seventh and eighth nerve	Carb Decompression	NA
2000	L.Tacconi et al. [40]	2	NA	2/0	2	2/0	19–24	NA	Carb Decompression	NA
1981	D. Testa et al. [41]	8	CMT1	6/2	3	2/1	35–46	Motor conduction times of the facial nerves were bilaterally prolonged	NA	NA
1978	S. K. Mongia et al. [42]	17	NA	9/8	4	NA	NA	NA	Nerve blocks Medical	NA
2019	Jean Loup Méreaux et al. [6]	8	CMT1B	3/5	4	0/4	30–51	Hearing loss	Carb, gaba, phenytoin, amitriptyline, clonazepam Thermocoagulation	MPZ
2019	James B. Caress et al. [16]	27	CMT1B	11/16	5	0/5	NA	Hemifacial spasm	NA	MPZ

*Carb* Carbamazepine, *Gaba* Gabapentine, *NA* Not available

The MARS1 mutations screened by our research were associated with CMT2u. The mutation, c.2398C > A p.(Pro800Thr), was also reported by Hyun et. in 2014, and it was associated with CMT2u [43]. This is the first study to identify potential FTN genes related to CMT type 2. However, CMT2u is a late-onset CMT (50–60 years of age), which is different from other subtypes. The average number of probands in Family 1 was 54.33 ± 4.16 years (mean ± SD). To date, our probands do not show symptoms of CMT. We will continue to follow up with Family 1.

In the future, further case series with available genetic data are needed to confirm the relationship between CMT and FTN. Further studies, including in vitro and in vivo experiments, are necessary to confirm our findings.

In addition, the missense MARS1 mutation c.2104C > T was first reported by our study in one proband from Family 3. but according to the ACMG/AMP variant classification, it is considered a VUS. Further experiments are needed to confirm its pathogenicity.

## Conclusion

Our study identified rare mutations in MARS1 that were compatible with FTN by using WES and Sanger sequencing. We analysed ISR-related genes and found that Fosl1 and NFE2 expression was significantly higher in the probands than that in TN patients. Combined with the results of a previous study, our results support that MARS1 mutations may cause chronic activation of the ISR, contribute to ISR pathophysiological changes and cause/accelerate peripheral nerve degeneration. Our study supports that FTN is a type of neurodegenerative disease. 

## Data Availability

All data and materials generated in this study are available upon request.

## Abbreviations

TN: Trigeminal neuralgia
FTN: Familial trigeminal neuralgia
WES: Whole-exome sequencing
MVD: Microvascular decompression
ISR: Integrated stress response
RNA-seq: Ribonucleic acid sequencing
